# Vascular Endothelial Growth Factor as a Potential Biomarker of Neuroinflammation and Frontal Cognitive Impairment in Patients with Alcohol Use Disorder

**DOI:** 10.3390/biomedicines10050947

**Published:** 2022-04-20

**Authors:** Nerea Requena-Ocaña, María Flores-Lopez, Esther Papaseit, Nuria García-Marchena, Juan Jesús Ruiz, Jesús Ortega-Pinazo, Antonia Serrano, Francisco Javier Pavón-Morón, Magí Farré, Juan Suarez, Fernando Rodríguez de Fonseca, Pedro Araos

**Affiliations:** 1 Laboratorio de Medicina Regenerativa (LMR), Unidad de Gestión Clínica de Salud Mental, Instituto de Investigación Biomédica de Málaga (IBIMA), Hospital Regional Universitario de Málaga, Avda. Carlos Haya 82, Sótano, 29010 Malaga, Spain; nerea.requena@ibima.eu (N.R.-O.); maria.flores@ibima.eu (M.F.-L.); ngarciam@igtp.cat (N.G.-M.); jesus.ortega@ibima.eu (J.O.-P.); antonia.serrano@ibima.eu (A.S.); javier.pavon@ibima.eu (F.J.P.-M.); 2 Departamento de Psicobiología, Facultad de Psicología, Universidad Complutense de Madrid, Campus de Somosaguas, 28040 Madrid, Spain; 3 Department of Clinical Pharmacology, Hospital Universitari Trias I Pujol and Institut de Recerca Germans Trias I Pujol (HUGTiP-IGTP), 08916 Badalona, Spain; epapaseit.germanstrias@gencat.cat (E.P.); mfarre.germanstrias@gencat.cat (M.F.); 4 Department of Pharmacology, Therapeutics and Toxicology, Universitat Autònoma de Barcelona, 08913 Cerdanyola del Vallès, Spain; 5 Institut D, Investigació en Ciències de la Salut Germans Trias i Pujol (IGTP), Unidad de Adicciones-Servicio de Medicina Interna, Campus Can Ruti, Carrer del Canyet s/n, 08916 Badalona, Spain; 6 Centro Provincial de Drogodependencias (CPD) de Málaga, Diputación de Málaga, C/Ana Solo de Zaldívar, n° 3, 29010 Malaga, Spain; jjruiz@malaga.es; 7 Instituto de Investigación Biomédica de Málaga (IBIMA), Unidad de Gestión Clínica del Corazón, Hospital Universitario Virgen de la Victoria de Málaga, Planta 5^a^-Sección Central, 29010 Malaga, Spain; 8 Centro de Investigación Biomédica en Red Enfermedades Cardiovasculares (CIBERCV), Instituto de Salud Carlos III, Calle de Melchor Fernández Almagro, 3, 28029 Madrid, Spain; 9 Department of Anatomy, Legal Medicine and History of Science, School of Medicine, University of Malaga, Boulevard Louis Pasteur 32, 29071 Malaga, Spain; 10 Departamento de Psicobiología y Metodología de las CC del Comportamiento, Facultad de Psicología, Universidad de Málaga, 29016 Malaga, Spain

**Keywords:** alcohol use disorders, addiction, VEGFA, blood–brain barrier, chemokines, fractalkine, neuroinflammation, cognitive dysfunction, neurodegeneration, dementia

## Abstract

(1) Background: Alcohol Use Disorder (AUD) is associated with functional disruption of several brain structures that may trigger cognitive dysfunction. One of the mechanisms of alcohol-associated cognitive impairment has been proposed to arise from its direct impact on the immune system, which culminates in the release of cytokines and chemokines which can eventually reach the brain. Alcohol can also disrupt the blood–brain barrier, facilitating the penetration of pro-inflammatory molecules throughout vascular endothelial growth factor A (VEGFA). Thus, alcohol-induced alterations in chemokines and VEGFA might contribute to the neuroinflammation and cognitive impairment associated with AUD. (2) Methods: The present cross-sectional study investigates whether patients with AUD (*n* = 86) present cognitive disability associated to alterations in plasma concentration of SDF-1, fractalkine, eotaxin, MCP-1, MIP-1α and VEGFA when compared to control subjects (*n* = 51). (3) Results: The analysis indicated that SDF-1 and MCP-1 concentrations were higher in AUD patients than in controls. Concentrations of VEGFA were higher in AUD patients with severe frontal deficits, and the score of frontal lobe functions was negatively correlated with VEGFA and fractalkine. Acute alcohol effects on VEGFA plasma levels in healthy volunteers demonstrated the induction of VEGFA release by heavy alcohol drinking. VEGFA was positively correlated with pro-inflammatory chemokines in AUD patients with frontal cognitive impairment. (4) Conclusions: we propose VEGFA/chemokine monitoring as biomarkers of potential cognitive impairment in AUD patients.

## 1. Introduction

Alcohol use disorder (AUD) is one of the main global health problems, carrying a significant social and economic burden. Alcohol abuse is responsible for more than 3 million deaths annually in the world, with the highest rates of alcohol consumption being in the European Union [1]. In Spain, alcohol is the most consumed drug among the general population (15–64 years): 91.2% at some time in their life, 75.2% in the last year, 62.7% in the last 30 days and 7.4% daily during the last month [2].

Among medical consequences related to AUD, we can highlight the induction of liver and pancreatic disease [3,4], psychiatric comorbidities, and other substance use disorders throughout life [5]. Major depressive disorders and anxiety disorders are the most prevalent comorbid psychiatric disorders, whereas cocaine and cannabis misuse are the most frequent comorbid substance use disorders associated with AUD [6,7]. Furthermore, a growing number of studies indicate that alcohol abuse is a major contributor to the development of any type of dementia, especially when there is an early onset of the cognitive impairment [8,9]. In addition, lifetime presence of chronic alcohol dependence has been suggested as an independent risk factor for the development of dementia. Thus, alcohol-related dementia (ARD) has been reported to be one the most prevalent, especially in young men (from 8.27 per 100,000 to 25.6% in several study populations) [10]. Moreover, heavy alcohol consumption has been related with a rapid progression of cognitive decline in aging [11]. According to these data, it is widely known that chronic alcohol consumption has a profound impact on brain structures that support higher cognitive functions [12,13,14].

Regarding molecular mechanisms mediating alcohol abuse-associated cognitive impairment, it is thought that it is derived from alcohol-induced neuroinflammation mediated by oxidative stress and by the release of proinflammatory signaling molecules (i.e., chemokines/cytokines), ultimately leading to neuronal apoptosis and even necrosis. This process may eventually lead to a permanent derangement of cognition, resulting in dementia. Increasing evidence supports an essential role for Toll-like receptors (TLRs) in alcohol-induced neurodegenerative disease [15]. Recent studies have observed that alcohol stimulates brain immune cells (microglia and astrocytes) by activating TLR (mainly TLR4) and NOD-like receptors. This activation culminates in the production of proinflammatory cytokines and chemokines, leading to neuroinflammation and neuronal damage in the cortex and the hippocampus. Hence, activation of TLR4 by ethanol triggers fast downstream signaling pathways such as mitogen-activated protein kinases (MAPKs) and nuclear factor-kappa B (NF-kB), eventually promoting the expression and release of cytokines, chemokines, and inflammatory mediators [16,17]. These events can be potentiated by alcohol-induced neuroinflammation inflammatory signals at peripheral tissues (i.e., gut, liver or pancreas) reaching the brain through ethanol’s disruption of the blood–brain barrier [18]. Thus, substantial evidence suggests that alcohol impacts the immune system and induces an up-regulation of cytokines and chemokines which are associated with behavioral changes and cognitive impairment [19,20].

Chemokines (chemotactic cytokines) are immune signals involved in cellular migration and intercellular communication. These proteins also act as modulators in neuronal transmission and contribute to communication between glia and neuronal cells [21,22]. In addition, cytokines are involved in the regulation of cell development, survival, and regeneration of the central nervous system [23,24]. These signals are important components of the neuroimmune system that contribute to neuronal activity, neuroendocrine function, brain development, synaptic plasticity, and circuity of mood in drug addiction [25,26]. Furthermore, chemokine decompensation described in plasma, serum and cerebrospinal fluid has been associated with several psychiatric and neurodegenerative diseases such as mild cognitive impairment, Alzheimer’s disease, Parkinson’s disease, schizophrenia, bipolar disorder and major depression [27,28,29,30]. However, despite the interaction between alcohol and immunological mediators having been well investigated [31,32], little is known about whether these immunoinflammatory signals impact the development of AUD-associated cognitive impairment. Long-lasting brain induction of the proinflammatory cytokines tumor necrosis factor alpha (TNFα), interleukin (IL)-1β and monocyte chemoattractant protein-1 (MCP-1) and the anti-inflammatory cytokine IL-10 have been related to microglial activation and reduced neurogenesis in mice exposed to LPS endotoxin after ethanol treatment [33]. However, plasma chemokines have been evaluated almost exclusively in the context of liver disease in alcohol-dependent patients [34,35].

On the other hand, Vascular Endothelial Growth Factor A (VEGFA) could be a potential candidate for explaining how alcohol facilitates both infiltration and inflammation in the brain. VEGFA is a protein that belongs to the family of growth factors and is commonly known for its role in angiogenesis and vascular permeability. In addition, VEGFA plays a fundamental role in the development and adult nervous system since it is involved in the extension and complexity of the microvasculature that supplies the necessary nutrients and oxygen in the brain. In this way, the effects of VEGFA on the nervous system have been related to neuroprotection, neurogenesis, and synaptic plasticity through the stimulation of neural stem cells and safeguarding the integrity of the blood–brain barrier [36]. Therefore, changes in VEGFA concentrations could affect the function and survival of neurons by not providing enough nutrients or producing hypoxia, which has been related to the deterioration of cognitive function [37]. Furthermore, VEGFA is a potent vasodilator and angiogenic factor released under hypoxic and stressful conditions via endothelial nitric oxide synthase [38,39]. Altered levels of VEGFA have been related to several neurodegenerative and neurological disorders, such as Alzheimer’s disease, vascular dementia and stroke [36]. Nevertheless, VEGFA is still poorly understood in the field of substance use disorders. Heberlein (2010) observed that VEGFA serum levels increase during alcohol withdrawal, and it might be intimately associated with alcohol intoxication and the severity of the addiction reflected by recurrent episodes of alcohol intoxication [40]. In addition, augmented VEGFA levels have been found in alcoholic liver disease patients compared to controls, showing a positive association with cholestatic enzymes [41].

Considering the previous antecedents, in the present study, we investigated the potential association of plasma concentrations of the chemokines stromal cell-derived factor 1 (SDF-1), fractalkine, eotaxin, MCP-1, macrophage inflammatory protein 1 alpha (MIP-1α) and the trophic factor VEGFA to frontal cognitive impairment in AUD patients. In addition, to fully understand the effects of alcohol on VEGFA, we studied the plasma concentration of this trophic factor after acute alcohol intake. The ultimate goal was to identify a potential link between AUD-associated cognitive impairment and plasma levels of VEGFA and chemokines that might be eventually useful for clinical purposes.

## 2. Materials and Methods

### 2.1. Recruitment and Screening of Participants

**Figure 1:**
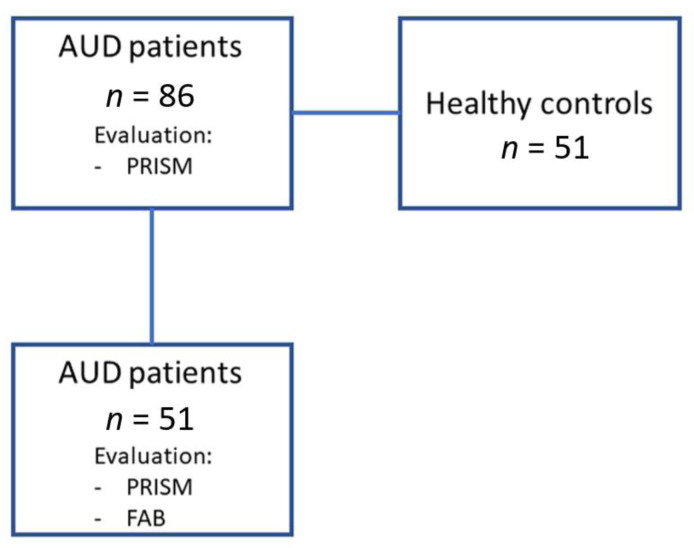
Diagram showing the design of the cross-sectional study.

The cross-sectional study included 137 Caucasian volunteers divided into two groups: 86 abstinent AUD patients (alcohol group) in outpatient treatment and 51 control subjects (control group) matched by age, body mass index (BMI) and proportion of sex. Patients were recruited at the Psychiatry Service of the Hospital Universitario 12 de Octubre (Madrid, Spain) and Centro Provincial de Drogodependencias (Málaga, Spain). Control participants were included from databases of healthy subjects (without presence of cognitive impairment, medical diseases and substance use disorders) of the Biobanco Nacional de DNA. In addition, we performed a brief frontal neuropsychological evaluation in 59% of AUD patients (Figure 1).

#### AUD Patients and Control Volunteers

To be included in the present study, participants had to meet the following inclusion criteria: people aged 18 to 65 years in the abstinence phase, being in outpatient treatment and willingness to participate by signing the informed consent. As we wanted to control for potential interferences in plasma concentrations of chemokines and VEGFA, the exclusion criteria included: use of anti-inflammatory drugs or MAOI’s, personal history of long-term inflammatory disease or cancer, pregnant or breast-feeding women and infectious diseases such as Hepatitis C, Hepatitis B and HIV.

An additional group of healthy subjects was recruited at Hospital German Trias I Pujol from Badalona, Spain, to investigate the acute actions of alcohol on VEGFA plasma concentrations. The study design was simple blind, non-randomized, non-controlled study of the experimental administration of alcohol simulating a “binge drinking” episode. Ten healthy male subjects were recruited and administrated an alcoholic beverage containing 100 g of alcohol (312 mL vodka Absolut^®^, Ahus, Sweden) were mixed with 588 mL of orange soda [Trina^®^ Orange No gas. Suntury Limited, Dōjimahama, Japan], total volume 900 mL. The alcoholic beverage was distributed in 6 identical glasses (volume 150 mL) and consumed continuously over a 2 h period (15 min per glass). Participants were selected after a general medical examination to exclude any psychopathological condition. Subjects signed an informed consent prior to participation and were economically compensated for any inconvenience caused during the trial. The participants had a mean age of 22 ± 2 years, mean weight 73.0 ± 9.2 kg, mean height 180 ± 6.5 cm and index body mass (IBM) 22.5 ± 1.9 kg/m^2^. They drank an average of 13.7 ± 8.3 g of alcohol per day and reported a mean 1.3 ± 1.7 alcohol binge episodes per month.

### 2.2. Ethical Statement

Written informed consent was obtained from each participant after a complete description of the study. All participants had the opportunity to discuss any questions or problems. For the cross-sectional study, the design and the recruitment protocols were approved by the Ethics Committee of the Hospital Regional Universitario de Málaga (PND 2019/040). The acute alcohol administration experiment protocol was approved by the local Human Research Ethics Committee (CEI Hospital Universitari Germans Trias i Pujol, Badalona, Spain) and registered at ClinicalTrials.gov (NCT02232789). All procedures were in strict accordance with the Ethical Principles for Medical Research with Human Subjects adopted in the Declaration of Helsinki by the World Medical Association (64th General Assembly of the WMA, Fortaleza, Brazil, October 2013) and Recommendation No. R (97) 5 of the Committee of Ministers to the Member States on the protection of medical data (1997), and the Spanish law on data protection [Regulation (EU) 2016/679 of the European Parliament and of the Council of 27 April 2016 on the protection of natural persons with regard to the processing of personal data and the free circulation of such data, and repealing Directive 95/46/EC (General Data Protection Regulation)]. All collected data received code numbers to maintain privacy and confidentiality.

### 2.3. Psychiatric and Neuropsychological Evaluation

The Spanish version of the PRISM (Psychiatric Research Interview for Substance and Mental Diseases) diagnostic interview was used for the evaluation of substance use disorders and other psychiatric disorders according to the criteria of the DSM-IV-TR (Diagnostic and Statistical Manual of Mental Disorders, 4th edition). The PRISM is a semi-structured interview with good psychometric properties in the evaluation of substance use disorders and in the main comorbid psychiatric disorders related to the substance use population [42,43].

The neuropsychological evaluation was performed using the Spanish version of the Frontal Assessment Battery (FAB) for the diagnosis related to frontal lobe dysfunctions [44] that have demonstrated reliability and good psychometric properties. The total FAB score was obtained from 0 to 18 evaluating the subdomains respectively: grasp, go-no-go, conflictive, lexical fluency, and motor skills. A cut-off point lower than 16 separates normal frontal deficits from mild ones, and a cut-off point lower than 13 separates mild and severe frontal syndrome.

### 2.4. Obtaining Plasma Samples

Blood samples were obtained in the morning after fasting for 8–12 h (before psychiatric interviews). Venous blood was extracted into 10 mL K2 EDTA tubes (BD, Franklin Lakes, NJ, USA) and immediately processed to obtain plasma. Blood samples are centrifuged at 2200 g for 15 min (4 °C) and individually tested for infectious diseases using 3 commercial rapid tests for HIV, Hepatitis B and Hepatitis C (Strasbourg, Cedex, France). Finally, the plasma samples were individually aliquoted, recorded and stored at −80 °C until further analysis.

### 2.5. Multiplexed Bead Immunoassay

Plasma concentrations of SDF-1, eotaxin, MIP-1, MCP-1, fractalkine and VEGFA were measured by using a human custom 7-ProcartaPlex bead immunoassay kit (Invitrogen, cat. no. PPX-07-MXH6ANW, Waltham, MA, USA) in a Luminex xMAP^®®^ technology—MAGPIG system (ThermoFisher, Waltham, MA, USA). Sensitivity was approximately 13, 33, 12, 51, 39 and 78 pg/mL for SDF-1, eotaxin, MIP-1α, MCP-1, fractalkine and VEGFA, respectively. Mean intra-assay variation (%CV replicates) was 5.3, 9.3, 10.3, 7.1, 11.1 and 12.1%, respectively, and mean inter-assay variation (%CV) was 29.7, 30.1, 44.6, 48.5, 36.7 and 19.9%, respectively, for all analyses. The minimum detectable concentration values were attributed to missing values that were under the standard curve.

### 2.6. Statistical Analysis

All data in tables are expressed as numbers and percentage of subjects [*n* (%)] or means and standard deviations (SD). The significance of the differences in the qualitative variables was determined through Fisher’s exact test (Chi-square). The normal distribution of the variables was assessed using Lilliefors corrected Kolmogorov-Smirnov test. For continuous variables that did not meet the assumption of normality, statistical analyses were performed using non-parametric Mann–Whitney *U*-test for comparisons between two groups and Spearman for correlations. For continuous variables that met the assumption of normality, we used an ANOVA with repeated measures. Lastly, a principal components analysis with varimax rotation and bivariate relationships (correlation) was performed to determine the different profiles of alcohol-abstinent patients with cognitive decline. Only variables with a factor load of at least 0.3 (i.e., those that share at least 10% of the variance with a factor) were used for the interpretation. A *p*-value less than 0.05 was considered statistically significant. Statistical analyses were carried out using GraphPad Prism version 5.04 and IBM SPSS Statistical version 22 (IBM, Armonk, NY, USA). For the time-course analysis of the acute effects of alcohol on VEGFA, ANOVA with repeated measures design was selected. In the case of plasma concentrations of VEGFA a non-parametric Friedman test for repeated measures was selected. A value of *p* < 0.05 was considered statistically significant.

## 3. Results

### 3.1. Sociodemographic Characteristics

Table 1 shows a socio-demographic description of the total sample. We selected 86 abstinent patients with AUD diagnosis and 51 healthy control subjects matched for sex, age and BMI. The mean age of the AUD group was 44 years and the 81% were men with a BMI index of 26. A significant difference was observed between the two sample groups when educational level and occupation was analyzed (*p* < 0.022, *p* < 0.001).

### 3.2. Alcohol-Related Variables in AUD Group

The variables related to the AUD group were evaluated and are described in Table 2. The mean age at first drink of alcohol was 15 years, while the average age of the AUD onset was 26 years with 15 years of problematic alcohol use. The mean of severity criteria of addiction was 8 (based on DSM-5) and they had a length of 322 days of abstinence at the moment of the evaluation.

Regarding other substance use disorders, tobacco (77%) and cocaine (48%) were the most prevalent drugs among AUD patients. In addition, an elevated prevalence of other comorbid psychiatric disorders was observed, with lifetime mood and anxiety disorders being the most frequently diagnosed, in 49% and 27%, respectively. Furthermore, 87% of the abstinent alcohol patients received psychiatric medication during the last year: anxiolytics (63%), antidepressants (52%), antipsychotics (11%) and anticraving (10%). Finally, 39% of the AUD group was treated with disulfiram.

The neuropsychological evaluation revealed that 55% of the AUD group showed some deficits related to frontal cognition (assessed by FAB): 45% did not have frontal deficits, 31% had mild cognitive deficits, and 23% showed severe cognitive impairment. We observed a high prevalence of sedative use disorders in patients without frontal cognitive impaired compared to patients with cognitive impairment (*U* = 5.180, *p* = 0.023). However, we did not find significant differences in other alcohol-related variables between patients with and without frontal cognitive impairment.

### 3.3. Plasma Concentrations of VEGFA and Chemokines in Abstinent AUD Patients

**Figure 2:**
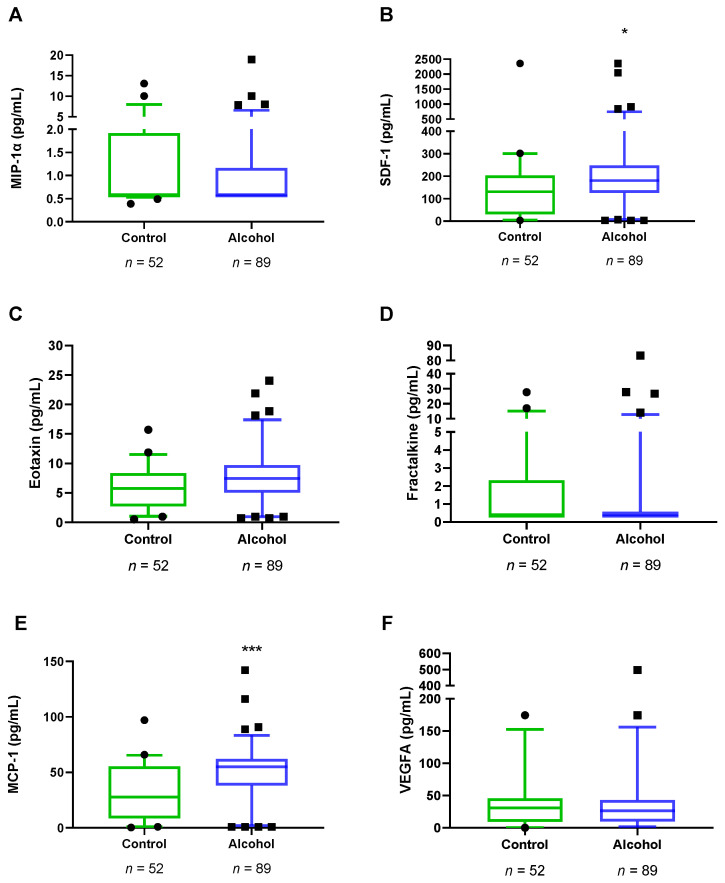
Plasma concentrations of VEGFA and chemokines in the alcohol group vs the control group (*n* = 141). (**A**) MIP-1α (pg/mL), (**B**) SDF-1 (pg/mL), (**C**) Eotaxin (pg/mL), (**D**) Fractalkine (pg/mL), (**E**) MCP-1 (pg/mL), and (**F**) VEGFA (pg/mL). Box and whiskers plotted at the 5–95 percentile. Dots are individual values. Data were analyzed by Mann–Whitney *U*-test. (*) *p* < 0.05 and (***) *p* < 0.001 denote significant differences compared with the control group.

The impact of alcohol dependence on plasma concentrations of VEGFA and chemokines was studied in the total sample using Mann–Whitney *U*-test. Plasma concentrations of SDF-1 (*U* = 1615, *p* = 0.010) and MCP-1 (*U* = 1354, *p* < 0.001) were significantly higher in the alcohol group compared to the control group (Figure 2). However, we did not observe major differences in MIP-1α, eotaxine, fractalkine and VEGFA between the alcohol group and the control group (Table S1).

Moreover, correlation analysis between plasma concentrations of VEGFA and chemokines and age at first alcohol use, age at onset of AUD, length of AUD diagnosis, severity criteria, and length of abstinence were conducted in these AUD patients. Plasma levels of SDF-1, eotaxin and VEGFA were found to be significantly correlated with alcohol addiction severity based on alcohol criteria (rho = 0.211, *p* < 0.048; rho = 0.250, *p* < 0.018; rho = 0.234, *p* < 0.027, respectively) (Table S2).

### 3.4. Plasma Concentrations of VEGFA and Chemokines in Abstinent AUD Patients with Frontal Cognitive Impairment

**Figure 3:**
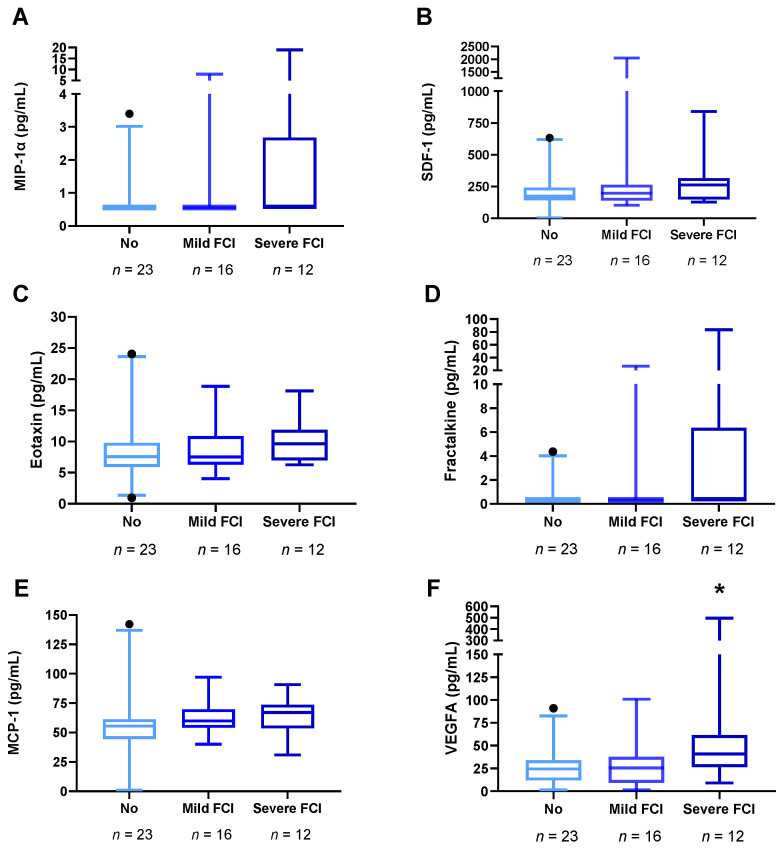
Plasma concentrations of VEGFA and chemokines in AUD patients with frontal cognitive impairment (*n* = 28). (**A**) MIP-1α (pg/mL), (**B**) SDF-1 (pg/mL), (**C**) Eo-taxin (pg/mL), (**D**) Fractalkine (pg/mL), (**E**) MCP-1 (pg/mL), and (**F**) VEGFA (pg/mL). Box and whiskers plotted at the 5–95 percentile. Dots are individual values. Data were analyzed by Kruskal Wallis test. (*) *p* < 0.05 denote a significant difference compared with AUD patients without frontal deficits. Abbreviations: FCI = Frontal Cognitive Impairment.

To explore the influence of frontal cognition integrity on plasma concentrations of VEGFA and chemokines, we performed a Kruskal Wallis test with “frontal cognitive impairment” (no, mild, and severe) as a factor. We did not find a significant effect of “frontal cognitive impairment” on plasma concentrations of VEGFA and chemokines (Table S3). However, as shown in Figure 3, circulant levels of VEGFA almost reached the significance (K = 5.404, *p* = 0.067), having higher plasma concentrations of VEGFA the AUD patients with severe frontal cognitive impairment than those without frontal deficits (*U* = 72.50, *p* = 0.021).

### 3.5. Correlation Analyses between Frontal Cognition and Plasma Concentrations of VEGFA and Chemokines in AUD Patients

For deeper analysis, we explored the relationship between frontal lobe functions (evaluated by FAB) and plasma concentrations of VEGFA and chemokines using Spearman correlations (rho). As shown in Table 3, there was a significant and negative correlation between FAB score and plasma concentrations of VEGFA (rho = −0.290, *p* = 0.039). We also observed a significant and negative correlation between FAB score and plasma concentrations of fractalkine (rho= −0.336, *p* = 0.016). However, we did not find significant correlations between FAB scores and the plasma concentrations of SDF-1, eotaxin, MIP-1α and MCP-1.

### 3.6. Correlation Analyses between Plasma Concentrations of VEGFA and Chemokines in AUD Patients with and without Mild Cognitive Impairment

**Figure 4:**
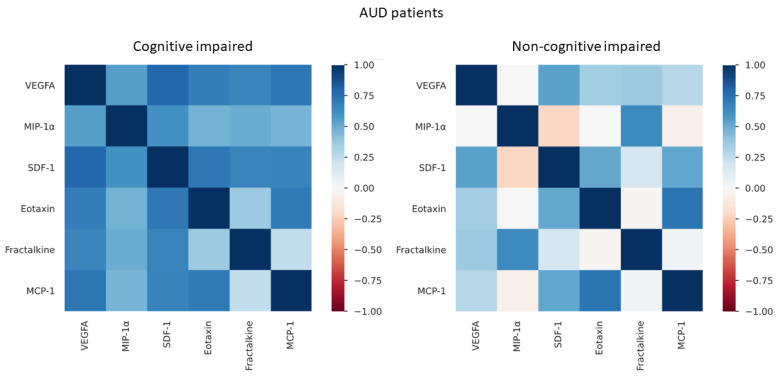
Correlations analysis between VEGFA and chemokines in AUD patients with and without frontal cognitive impairment (*n* = 51). Colors show Spearman’s rho correlation coefficient.

Moreover, we wanted to explore the relationship between plasma concentrations of chemokines and VEGFA depending on the integrity of frontal lobe function (evaluated by FAB) using Spearman correlations (rho). As shown in Figure 4, AUD patients with cognitive impairment displayed strong positive and significant correlations between VEGFA with all chemokines [SDF-1 (rho = 0.787, *p* < 0.001), eotaxin (rho = 0.678, *p* < 0.001), MIP-1α (rho = 0.592, *p* = 0.001), MCP-1 (rho = 0.601, *p* = 0.001), fractalkine (rho = 0.706, *p* < 0.001)], while we only observed a positive and significant correlation between VEGFA and SFD-1 (rho = 0.532, *p* = 0.009) for AUD patients without cognitive impairment (Table S4).

### 3.7. Differential Profiles Associated with VEGFA and Chemokines in AUD Patients with Frontal Cognitive Impairment

**Figure 5:**
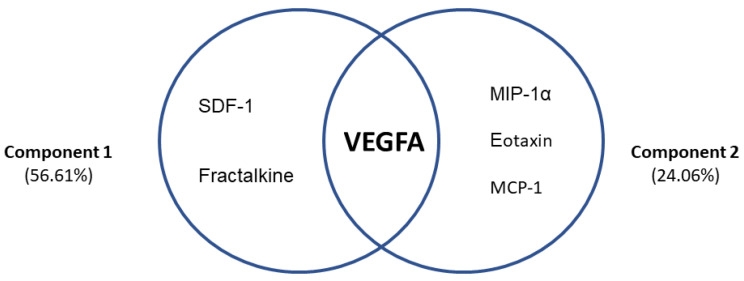
Exploratory principal component analysis in AUD patients with frontal deficits (*n* = 51). Two components together explained 80.67% of the variance associated with frontal cognitive impairment in AUD patients.

To understand the contribution of VEGFA and chemokines in the frontal cognitive decline of AUD patients, a principal component analysis was performed. Two components together explained 80.67% of the variance associated with cognitive impairment in AUD patients (Figure 5). Component 1 explained 56.61% of the total variance and was composed of SDF-1, fractalkine and VEGFA, which had high factor loads (0.984, 0.983, 0.757, respectively). Component 2 explained 24.06% of the total variance and was composed of MIP-1α, eotaxin, MCP-1 and VEGFA, which had high factor loads (0.669, 0.904, 0.807, 0.582, respectively).

### 3.8. Plasma Concentrations of VEGFA and Chemokines in Comorbid Medical, Psychiatric Medication and Substance Use Problems in AUD Patients

We wanted to investigate whether medical and psychiatric comorbidity, other concomitant substance use disorder or using psychotropic medication could affect plasma concentrations of VEGFA and chemokines in the alcohol group. Plasma concentrations of MIP-1α (*U* = 567, *p* = 0.002) and VEGFA (*U* = 552, *p* = 0.005) were significantly higher in AUD patients with comorbid psychiatric disorder compared to those without psychiatric comorbidity (Table 4). Moreover, plasma concentrations of MIP-1α (*U* = 633, *p* = 0.005) were significantly higher in AUD patients with comorbid substance use disorder compared to those without comorbid substance use disorder (Table S5). Regarding comorbid medical problems and the use of psychotropic medication, we did not observe major differences in plasma levels of chemokines and VEGFA (Tables S6 and S7).

### 3.9. Time Course of Plasma Concentrations of VEGFA after an Acute Administration of Alcohol (100 g) in Healthy Male Volunteers

**Figure 6:**
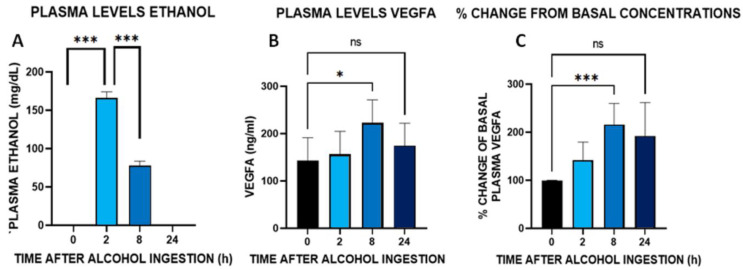
Plasma concentrations of alcohol (**A**) and VEGFA (**B**) at 2, 8 and 24 h after alcohol (100 g) ingestion in male (*n* = 9–10) healthy volunteers. 0 represents the time of ingestion. Alcohol resulted in increase in plasma concentrations of VEGF 8 h after its ingestion (Friedman’s non-parametric test for repeated measures, * *p* < 0.05 versus 0 time). (**C**) Percentage of change of VGEFA calculated over basal concentrations (ANOVA with repeated measures, *** *p* < 0.001 versus 0 time). ns = non-significant.

To clarify how an acute intoxicating dose of alcohol affects circulating levels of VEFGA, we administered 100 g of alcohol to male healthy volunteers whose daily average alcohol intake was of 13.7 ± 8.3. Plasma samples were taken previous to (time 0) and 2, 8, and 24 h after oral ingestion of alcohol. As expected, plasma ethanol level peaked at 2 h after ingestion and decreased to a third of the 2 h concentration 8 h after the ingestion, being undetectable 24 h after oral intake (Figure 6A). Levels of VEGFA were very variable in between these subjects so a non-parametric statistics with repeated measures approach was taken. Data indicated that alcohol modified plasma VEGFA, being elevated 8 h (Figure 6B) after the ingestion of alcohol (Friedman’s statistic for *n* = 9, four groups, was 9.26, *p* < 0.03). Analysis of the percentage of change from basal values indicated the alcohol induced a 2-fold change on VEGFA circulating concentrations with respect to the basal values (Figure 6C, ANOVA repeated measures F (8,24) = 5.14, *p* < 0.001). Twenty-four hours after the intake of alcohol, the % of change had a non-significative average fold change of 1.7, suggesting that alcohol induced sustained increases of VEGFA. However, this assumption needs to be conclusively determined.

## 4. Discussion

While the association between immune signaling and neuropsychiatric disorders has been widely investigated, its link with AUD-related cognitive impairment remains poorly investigated. We lack relevant information concerning how immune mediator-induced proinflammatory states in the brain influence the neuroadaptations derived from chronic alcohol consumption, and how they impact executive functions. In the present study, we unveil a link between plasma concentrations of VEGFA and chemokines with frontal lobe dysfunction in abstinent AUD patients treated in an outpatient setting. The relevance of the present study rests on the confirmation of the link between alcohol-induced dysfunctions in modulators of the blood–brain barrier (i.e., VEGFA) and neuroinflammation (i.e., chemokines) with the presence of cognitive impairment. The main findings are as follows: (i) AUD patients had increased plasma concentrations of SDF-1 and MCP-1 compared to control subjects; (ii) there were higher circulant levels of VEGFA in AUD patients with severe frontal deficits than in those without cognitive impairment; (iii) acute administration of a heavy dose of VEGFA resulted in a delayed increase (8 h after alcohol ingestion) of plasma VEGFA concentration; (iv) the integrity of frontal lobe functions was negatively correlated with VEGFA and fractalkine; (v) plasma concentrations of VEGFA were strongly and positively correlated with all chemokines in AUD patients with frontal deficits but not in those without frontal impairment; (vi) two components together explained 80.67% of the variance associated with frontal deficits in AUD patients, with VEGFA acting as a link between all chemokines; and (vii) plasma concentrations of some chemokines changed in AUD patients with comorbid psychiatric (MIP-1α, VEGFA) and substance use (MIP-1 α) disorders.

A growing literature indicates that the pharmacodynamic action of several drugs involves changes in the neuroimmune signal [45]. Some studies have reported that alcohol-related behaviors are interrupted when the innate immune network is disturbed [46,47]. Thus, Blednov et al. (2005) showed that gene deletion of CCR2, MCP-1 or MIP-1α reduced motivational effects of alcohol consumption in mice [48]. Moreover, Steinar et al. (2019) found an increase in the plasma concentrations of IL-6, IFNγ and MCP-1 in patients with a history of chronic alcohol overconsumption [49]. In agreement with these reports, the AUD patients of the present study displayed higher plasma concentrations of SDF-1 and MCP-1 than control subjects. SDF-1 was also associated (along with eotaxin) with worse severity of alcohol addiction. Even post mortem analysis reported increased expression of MCP-1 in multiple limbic brain regions in alcoholic subjects [50]. Taking into account the high prevalence of cocaine use disorder in AUD patients, it is important to note that extensive preclinical research has reported how the chemokines SDF-1 and MCP-1 promote cocaine-related behaviors in a brain region specific manner [51,52,53]. Our group has described that severity of cocaine consumption is related to IL-1β, SDF-1 and fractalkine in cocaine use disorder patients [54]. Furthermore, we found higher plasma concentrations of fractalkine in abstinent cocaine patients with comorbid major depressive disorder than in those without this psychiatric condition [55]. In addition, we demonstrated the induction of a potent fractalkine signaling associated with cocaine-induced sensitization and extinction in mice [56].

Regarding cognitive function, 55% of the AUD patients recruited in the present study displayed some kind of frontal cognitive impairment. Accordingly, executive functions are particularly affected in AUD patients who show deficits in domains related to cognitive control, flexibility, inhibition, planning and working memory [57,58]. However, there are other neuropsychological processes that could be disrupted, including memory, emotion, and social cognition [59]. It is important to note that despite there being evidence of partial recovery of certain cognitive functions after cessation of alcohol intake [13,60], deficits in other domains may remain stable during sobriety [57,61]. Additionally, cognitive impairment can compromise efforts to initiate and maintain abstinence by impacting treatment effectiveness [62]. It is thought that increased vulnerability to alcohol-induced working memory impairment may impact in the ability to moderate alcohol consumption [63] and cognitive training could reduce the number of beverages in these patients [64]. Furthermore, our results indicate that AUD patients have low educational and occupational attainment, which has been related to worse neuropsychological performance, more indicators of neurocognitive disorders, early drug use onset and development of addiction, high-severity substance-related problems, and worse treatment outcomes [65,66,67]. Thus, excessive alcohol consumption has been linked to worse cognitive functioning in patients with low socioeconomic status operated by educational and occupational achievements [68]. Moreover, a longitudinal study has suggested that an educational level lower than high school and a low job occupation is associated with an increased risk of dementia in alcohol patients [69]. Similarly, our group has recently found that a high educational level could play a protective role in the onset, development, and progression of cocaine use disorders and could also protect against cognitive impairment caused by alcohol consumption throughout life [70,71]. Interestingly, in this study we found a negative association between the state of frontal lobe functions with two signaling molecules, VEGFA and fractalkine, involved in vascular function and neural plasticity.

VEGFA has been related to neuroprotection, neurogenesis, and synaptic plasticity mechanisms in the central nervous system through stimulation of neuronal stem cells and safeguarding the integrity of the blood–brain barrier [36]. Despite VEGFA levels having been linked to several neurodegenerative and neurological disorders [36], their effects could be time dependent. Augmented VEGFA levels in cerebrospinal fluid and plasma have been reported in Alzheimer’s disease and vascular dementia patients [72,73] probably as a consequence of hypoperfusion and hypoxia. Nevertheless, improvement in learning and memory after a bilateral carotid artery occlusion has been associated with an increase in VEGFA expression in the hippocampus, which suggests that VEGFA signaling could compensate for cognitive impairment [74,75]. Similarly, partial increases in VEGFA stimulate vasodilation, angiogenesis and neuroprotection mechanisms, which are beneficial for the brain in later stages after cerebral ischemia [76]. However, early VEGFA increases may lead to undesirable effects in cerebral ischemia, such as an increase in blood–brain barrier permeability and infiltration of immune cells inducing neuroinflammation and edema [77,78,79].

With reference to the latter, in the present study we observed that increases in VEGFA were associated with worse severity of alcohol addiction, severe frontal deficits and the elevation of all chemokines in frontal cognitive impaired AUD patients. Interestingly, in a pilot study with healthy male volunteers with a history of moderate alcohol consumption, we found that acute alcohol administration resulted in a delayed increase in plasma VEGFA, observed 8 h after alcohol intake. Because of the increased vascular permeability induced by VEGFA, it is reasonable to think that this action of alcohol might facilitate neuroinflammation by opening the blood–brain barrier to pro-inflammatory signals originating in peripheral tissues, especially in the intestine. Supporting this hypothesis, in our principal component analysis, we found that the interaction of chemokines and VEGFA explained the 80.67% of the variance associated with frontal deficits in AUD patients, observing that VEGFA has an essential role as a factor interacting with the pro-inflammatory immune response associated with alcohol consumption. Consistent with our results, using cellular and animal models, Muneer et al. (2012) found that chronic alcohol exposure disrupts the blood–brain barrier across degradation of endothelial VEGF receptor 2. This also increases circulating levels of VEGFA leading to neuronal death and inflammation in the brain [80]. Moreover, Louboutin et al. (2012) reported increases in VEGFA levels and blood vessel density in cerebral tissue within two weeks of the onset of ethanol consumption in rats [81]. Moreover, despite VEGFA not being an inflammatory cytokine, it can activate nuclear factor-enhancer of activated B-cell kappa light chains (NF-KB) and nuclear factor of activated T-cells (NFAT) signaling cascades that could promote a chemotaxis response involved in the angiogenic process (although this role remains unknown) [82]. Thus, our results in AUD patients with frontal deficits might suggest two things: (1) chronic alcohol abuse might lead to alterations in the concentrations of VEGFA that increase the permeability of the blood–brain barrier, leading to infiltration of immune cells and inflammation in the brain [80,81], and/or (2) under the presence of hypoperfusion and hypoxia as a result of alcohol-derived brain damage, concentrations of VEGFA might ultimately increase as a compensatory signal in order to form new blood vessels and recruit chemokines to the affected brain area. Lastly, it has been found that higher circulant levels of VEGFA in major depression and its alterations are related to impaired cognitive function in schizophrenia [83,84]. This might explain why psychiatric comorbidity affected plasma concentrations of VEGFA in our study. It is important to note that cognitive deficits found in patients with addictions can often be exacerbated by comorbid psychiatric disorders [85].

Lastly, previous studies have reported that fractalkine (CX3CL1) develops an essential role in the neuronal–microglial intercommunication [86]. This chemokine is expressed from brain neurons that control activation of microglia through its binding receptor CX3CR1. In harmful conditions, neurons release fractalkine in order to stimulate proliferation, activation and migration at the site of the brain injury [87,88]. CX3CL1-KO mice showed altered microglial function and neurotoxicity following LPS injection as well as more neuronal damage in Parkinson’s disease and amyotrophic lateral sclerosis [88]. In accordance with this, Sokolowki et al. (2014) found that CX3CL1-KO mice revealed signals of neuronal apoptosis after ethanol treatment, suggesting a role in the clearance of those apoptotic cells [86]. Moreover, additional studies have reported that mild–moderate Alzheimer’s disease patients had higher plasma levels of CX3CL1 than those with severe Alzheimer’s disease [89,90]. These results suggest that fractalkine and CX3CR1 signaling might act as a neuroprotective mechanism through the microglial activity modulation in the early stage of brain injury while this signal seems to disappear when neuronal damage is established [91]. This may indicate that the AUD patients in this study are actively fighting against cognitive impairment.

## 5. Conclusions, Limitations, and Future Perspectives

In conclusion, a lifetime of chronic alcohol consumption leads to a proinflammatory systemic condition revealed by enhanced circulating chemokines, and to frontal cognitive impairment. The trophic factor VEGFA appears to be a relevant contributor to alcohol-associated neuroinflammation, probably through its role on controlling blood–brain barrier permeability, ultimately leading to impaired cognition. In addition, fractalkine could act as a signal of brain damage in early stages of cognitive impairment. Potential biomarkers could be useful and reliable tools in patients with AUD for confirming the diagnosis, defining the current stage of the AUD, and diagnosing these patients early.

Nevertheless, this study has several limitations that should be taken into account in future research. First, we do not know the time course of the effects of alcohol on these chemokines, either after acute or chronic alcohol consumption, nor its alterations in early or extended abstinence. We must also investigate whether sociodemographic variables, especially educational level, time of alcohol consumption, age of alcohol drinking initiation, etc., might contribute to the cognitive performance in both control and AUD patients. Finally, we lack significant representation of the female population, which precludes investigation of sex differences in chemokines and VEGFA. However, the data obtained clearly point to the need of considering these immunoinflammatory signals and trophic factors as relevant biomarkers of AUD-associated complications. Moreover, this concept should be extended to the analysis of immunomodulators capable of activating chronic inflammation. There are several biochemical pathways affected by alcohol consumption that need to be considered under the light of the present discoveries. For example, the tryptophan/kynurenine pathway is a potent immunomodulatory system that can modify inflammation, learning and memory [92,93]. The intestinal microbiota (which is determinant for AUD) [94] participates in these pathways, modifying the presence of pro- and anti-inflammatory mediators and eventually growth factors.

As a future perspective, we need to integrate all the information related to this multiplicity of inflammatory signals in a single model of alcohol addiction. Although certain factors such as VEGFA or fractalkine may contribute to important aspects of alcohol addiction, the complexity of the interactions of these inflammatory signaling proteins goes beyond our current technique and knowledge. Further clinical and technological research is necessary to elucidate the role of these factors in the etiology of AUD and associated comorbidities.

## Figures and Tables

**Table 1:** Socio-demographic characteristics of the total sample.

	Total Sample (*n* = 141)				
Variables		Control Group (*n* = 52)	Alcohol Group (*n* = 89)	Statistic	*p*-Value
Age (Mean ± SD)	Years	47.14 ± 5.29	44.16 ± 11.88	1867.50 ^1^	0.056
Body Mass Index (Mean ± SD)	Kg/m^2^	27.15 ± 3.59	26.36 ± 4.84	1907.50 ^1^	0.117
Sex [*n* (%)]	Women Men	17 (32.70) 35 (67.30)	17 (19.10) 72 (80.90)	3.313 ^2^	0.069
Education Degree [*n* (%)]	Elementary Secondary University	13 (25) 20 (38.50) 19 (36.50)	36 (40.40) 38 (42.70) 15 (16.90)	7.672 ^2^	**0.022**
Occupation [*n* (%)]	Employed Unemployed Retired Other	45 (86.50) 0 3 (5.80) 4 (7.70)	19 (21.30) 39 (43.80) 13 (14.60) 18 (20.20)	59.081 ^2^	**<0.001**

^1^ Value was calculated with Mann–Whitney *U*-test. ^2^ Value was calculated with Fischer’s exact test. Bold values are statistically significant for *p* < 0.05.

**Table 2:** Clinical characteristics of AUD patients with and without frontal cognitive impairment.

Variables		AUD Group				
		Total AUD (*n* = 89)	AUD with FCI (*n* = 28)	AUD without FCI (*n* = 23)	Statistic	*p*-Value
Age at first alcohol use (Mean ± SD)	Years	14.69 ± 4.027	14.42 ± 3.62	15.62 ± 3.71	271 ^1^	0.330
Age at onset of AUD (Mean ± SD)	Years	25.99 ± 9.591	28.38 ± 12.31	26.20 ± 9.58	266.50 ^1^	0.417
Length of AUD diagnosis (Mean ± SD)	Years	15.06 ± 11.314	11.46 ± 8.96	15.19 ± 11.40	228 ^1^	0.334
Severity criteria (Mean ± SD)	Criteria [1,2,3,4,5,6,7,8,9,10,11]	8.09 ± 2.114	7.96 ± 2.20	8.52 ± 2.32	299.50 ^1^	0.666
Length of abstinence (Mean ± SD)	Days	322.12 ± 908.545	63.46 ± 60.69	432.95 ± 1069.93	305.50 ^1^	0.961
Comorbid substance use disorders [*n* (%)]	Tobacco Cocaine Cannabis Sedatives	69 (77.50) 43 (48.30) 19 (21.30) 7 (7.90)	21 (75) 12 (42.90) 4 (14.30) -	21 (91.30) 11 (47.80) 4 (17.40) 4 (17.40)	2.264 ^2^ 0.126 0.090 5.180	0.132 0.723 0.764 0.023
Comorbid psychiatric disorders [*n* (%)]	Mood Anxiety ADHD Personality Psychotic	44 (49.4) 24 (27) 19 (21.30) 14 (15.70) 8 (9)	14 (50) 6 (21.40) 3 (10.70) 5 (17.90) 3 (10.70)	8 (34.80) 5 (21.70) 2 (8.70) 4 (17.40) 1 (4.30)	1.192 ^2^ 0.001 0.057 0.002 0.694	0.275 0.979 0.811 0.966 0.405
Psychiatric medication [*n* (%)]	Antidepressants Anxiolytics Antipsychotics Disulfiram Anticraving	46 (51.70) 56 (62.90) 10 (11.20) 35 (39.30) 9 (10.10)	17 (60.70) 15 (53.60) 2 (7.10) 14 (50) 5 (17.90)	12 (52.20) 18 (78.30) 1 (4.30) 10 (43.50) 1 (4.30)	0.375 ^2^ 3.370 0.175 0.216 2.117	0.540 0.066 0.676 0.642 0.204

Abbreviations: FCI = Frontal Cognitive Impairment, ADHD = attention deficit hyperactivity disorder (childhood). ^1^ Value was calculated with Mann–Whitney *U*-test. ^2^ Value was calculated with Fischer’s exact test. Bold values are statistically significant for *p* < 0.05.

**Table 3:** Correlation analysis between FAB scores and plasma concentrations of chemokines and VEGFA in AUD patients (*n* = 51). (rho) Spearman’s correlation coefficient. Bold values are statistically significant for *p* < 0.05.

Variables	FAB (Score)	
	Rho	*p*-Value
SDF-1 (pg/mL)	−0.228	0.111
Eotaxin (pg/mL)	−0.147	0.303
MIP-1α (pg/mL)	−0.154	0.280
MCP-1 (pg/mL)	−0.203	0.152
Fractalkine (pg/mL)	−0.336	**0.016**
VEGFA (pg/mL)	−0.290	**0.039**

**Table 4:** Plasma concentrations of chemokines and VEGFA grouped according to comorbid psychiatric disorder. Bold values are statistically significant for *p* < 0.05.

AUD Group (*n* = 89)				
Variables	Comorbid Psychiatric Disorder (*n* = 60)	No Comorbid Psychiatric Disorder (*n* = 29)	Statistics	
			*U*-Value	*p*-Value
	Mean [95% CI]	Mean [95% CI]		
SDF-1 (pg/mL)	271.9046 [165.9141–377.8952]	189.3975 [143.9530–234.8421]	831	0.828
Eotaxin (pg/mL)	7.77278 [6.57250–8.97306]	7.47531 [5.57394–9.37668]	806	0.575
MIP-1α (pg/mL)	1.8189 [1.0330–2.6048]	0.6189 [0.5586–0.6793]	567	**0.002**
MCP-1 (pg/mL)	48.9765 [42.4613–55.4917]	48.4279 [36.5812–60.2747]	813	0.618
Fractalkine (pg/mL)	2.0546 [0.6521–3.4570]	0.7399 [0.1778–1.3020]	741	0.160
VEGFA (pg/mL)	36.4530 [28.3181–44.5879]	20.1710 [14.3319–26.0100]	552	**0.005**

## Data Availability

Not applicable.

## Supplementary Materials

The following supporting information can be downloaded at: https://www.mdpi.com/article/10.3390/biomedicines10050947/s1, Table S1: Plasma concentrations of chemokines and VEGFA in AUD group versus control subjects; Table S2: Correlation analysis between FAB score and chemokines in AUD patients; Table S3: Correlation analysis between plasma concentrations of chemokines and VEGFA in AUD patients with and without cognitive impairment; Table S4: Plasma concentrations of chemokines and VEGFA grouped according to comorbid psychiatric disorder; Table S5: Plasma concentrations of chemokines and VEGFA grouped according to comorbid substance use disorder; Table S6: Plasma concentrations of chemokines and VEGFA grouped according to comorbid medical problem; Table S7: Plasma concentrations of chemokines and VEGFA grouped according to the use of psychotropic medication last year.
