# Supplementary material for: Vascular Endothelial Growth Factor as a Potential Biomarker of Neuroinflammation and Frontal Cognitive Impairment in Patients with Alcohol Use Disorder
Source: Biomedicines. 2022 Apr 20;10(5):947. doi: 10.3390/biomedicines10050947 (PMC9138236; doi:10.3390/biomedicines10050947)
Supplement: Supplementary file 1 [file biomedicines-10-00947-s001.zip › biomedicines-1633305-supplementary.pdf]

biomedicines

Supplementary Materials

Table S1. Plasma concentrations of chemokines and VEGFA in AUD group versus control subjects. Bold values are statistically significant for p<0.05.

VARIABLES	Total Sample (N=141)			
	Control group (N=52)	Alcohol group (N=89)	Statistics	
	Mean [95%CI]	Mean [95%CI]	U-value	*p*-value
**SDF-1 (pg/ml)**	178.8410 [70.0888-287.5932]	244.7148 [172.4289-317.0007]	1615.50	**0.010**
**Eotaxin (pg/ml)**	6.15676 [5.06444-7.24908]	7.67475 [6.67636-8.67314]	1874.50	0.060
**MIP-1α (pg/ml)**	1.5314 [0.9254-2.1374]	1.4234 [0.8877-1.9592]	2066	0.424
**MCP-1 (pg/ml)**	32.7873 [25.4970-40.0776]	48.7957 [43.0752-54.5162]	1354.50	**<0.001**
**Fractalkine (pg/ml)**	1.5916 [0.1812-3.0020]	1.6213 [0.6644-2.5782]	2176.50	0.790
**VEGFA (pg/ml)**	33.7697 [24.1271-43.4123]	31.0873 [25.1521-37.0226]	2176.50	0.831

Table S2. Correlation analysis between plasma concentrations of chemokines and VEGFA and alcohol-related variables.

VARIABLES	Alcohol-related variables				
	Age at first alcohol use (years)	Age at onset of AUD (years)	Length of AUD diagnosis (years)	Severity criteria (1-11)	Length of abstinence (days)
**SDF-1 (pg/ml) [Rho, *p* value]**	0.080 (0.460)	0.097 (0.369)	-0.138 (0.209)	**0.211 (0.048)**	0.143 (0.186)
**Eotaxin (pg/ml) [Rho, *p* value]**	0.126 (0,241)	0.182 (0.090)	0.076 (0.487)	**0.250 (0.018)**	0.119 (0.271)
**MIP-1α (pg/ml) [Rho, *p* value]**	-0.148 (0.165)	0.026 (0.812)	-0.138 (0.206)	-0.160 (0.133)	0.048 (0.659)
**MCP-1 (pg/ml) [Rho, *p* value]**	0.033 (0.762)	0.067 (0.532)	-0.102 (0.354)	0.184 (0.084)	0.185 (0.084)
**Fractalkine (pg/ml) [Rho, *p* value]**	-0.148 (0.168)	-0.074 (0.493)	0.050 (0.651)	-0.146 (0.173)	0.056 (0.602)
**VEGFA**	-0.121 (0.258)	0.050 (0.645)	-0.025 (0.823)	**0.234 (0.027)**	0.001 (0.989)

Table S3. Plasma concentrations of chemokines and VEGFA in AUD patients with and without frontal cognitive impairment. Abbreviations: FCI=Frontal Cognitive Impairment.

VARIABLES	AUD group (N=51)				
	AUD without FCI (N=23)	AUD with mild FCI (N=16)	AUD with severe FCI (N=12)	Statistics	
				K-value	*p*-value

	Mean [95%CI]	Mean [95%CI]	Mean [95%CI]		
**SDF-1 (pg/ml)**	**202.9657 [141.7296-64.2018]**	351.8619 [90.4870-613.2367	302.6091 [158.9163-446.3018]	2.766	0.251
**Eotaxin (pg/ml)**	8.64726 [6.36609-10.92843]	9.15286 [6.86654-11.43918]	10.28048 [7.62424-12.93673]	2.926	0.231
**MIP-1α (pg/ml)**	0.7710 [0.5016-1.0403]	1.0681 [0.0950-2.0412]	2.7650 [-0.9282-6.4582]	2.526	0.283
**MCP-1 (pg/ml)**	53.9017 [39.8406-67.9628]	62.6028 [54.8435-70.3621]	65.4501 [55.1646-75.7355]	4.025	0.134
**Fractalkine (pg/ml)**	0.6019 [0.1912-1.0125]	2.8346 [-1.0167-6.6860]	2.1818 [-0.3952-4.7588]	3.795	0.150
**VEGFA (pg/ml)**	24.8196 [16.2157-33.4236]	31.5217 [15.5495-47.4940]	39.0283 [25.7315-52.3252]	5.404	0.067

Table S4. Correlation analysis between plasma concentrations of chemokines and VEGFA in AUD patients with and without cognitive impairment. Bold values are statistically significant for p<0.05.

**VARIABLES**	**VEGFA**			
	**Frontal cognitive impairment N=30**	***p*-value**	**No frontal cognitive impairment N=20**	**p-value**
**SDF-1 [Rho]**	0.787	**<0.001**	0.532	**0.009**
**Eotaxin [Rho]**	0.678	**<0.001**	0.344	0.108
**MIP-1α [Rho]**	0.592	**0.001**	0.001	0.997
**MCP-1 [Rho]**	0.601	**0.001**	0.284	0.189
**Fractalkine [Rho]**	0.706	**<0.001**	0.344	0.108

Table S5. Plasma concentrations of chemokines and VEGFA grouped according to comorbid substance use disorder. Bold values are statistically significant for p<0.05.

	**AUD group (N=86)**			
**VARIABLES**	**Comorbid substance use disorder (N=46)**	**No comorbid substance use disorder (N=42)**	**Statistics**	
	**Mean [95%CI]**	**Mean [95%CI]**	**U-value**	***p*-value**
**SDF-1 (pg/ml)**	234.4558 [126.6838-342.2278]	255.9508 [156.3400-355.5617]	798	0.326
**Eotaxin (pg/ml)**	7.06242 [5.53708-8.58776]	8.34540 7.05016-9.64064]	706	0.092
**MIP-1α (pg/ml)**	1.6395 [0.9978-2.2813]	1.1868 [0.2873-2.0862]	633	**0.005**
**MCP-1 (pg/ml)**	46.7354	51.0522	888.50	0.964

	[37.3314-56.1394]	[44.5444-57.5601]		
**Fractalkine (pg/ml)**	1.5224 [0.2302-2.8146]	1.7297 [0.2523-3.2071]	738	0.088
**VEGFA (pg/ml)**	30.2696 [21.0863-39.4528]	31.9830 [24.2616-39.7044]	855.50	0.568

Table S6. Plasma concentrations of chemokines and VEGFA grouped according to comorbid medical problem.

**VARIABLES**	**AUD group (N=89)**			
	**Comorbid medical problem (N=26)**	**No comorbid medical problem (N=63)**	**Statistics**	
	**Mean [95%CI]**	**Mean [95%CI]**	**U-value**	***p*-value**
**SDF-1 (pg/ml)**	195.5504 [121.2791-269.8217]	264.2245 [166.8693-361.5796]	765	0.835
**Eotaxin (pg/ml)**	8.37289 [6.30432-10.44146]	7.39771 [6.23919-8.55622]	645	0.116
**MIP-1α (pg/ml)**	1.1645 [0.5433-1.7856]	1.5262 [0.8114-2.2410]	714	0.403
**MCP-1 (pg/ml)**	49.7528 [38.2961-61.2096]	48.4159 [41.6388-55.1931]	803.50	0.889
**Fractalkine (pg/ml)**	1.1624 [-0.0010-2.3257]	1.8035 [0.5335-3.0734]	755	0.472
**VEGFA (pg/ml)**	33.9870 [23.5237-44.4503]	29.9367 [22.6043-37.2691]	663.50	0.160

Table S7. Plasma concentrations of chemokines and VEGFA grouped according to the use of psychotropic medication last year.

**VARIABLES**	**AUD group (N=86)**			
	**Psychotropic medication (N=77)**	**No psychotropic medication (N=12)**	**Statistics**	
	**Mean [95%CI]**	**Mean [95%CI]**	**U-value**	***p*-value**
**SDF-1 (pg/ml)**	258.2095 [175.1311-341.2879]	159.2482 [95.3644-223.1320]	410	0.576
**Eotaxin (pg/ml)**	7.66472 [6.58220-8.74724]	7.73829 [4.71842-10.75816]	422	0.631
**MIP-1α (pg/ml)**	1.4755 [0.8610-2.0901]	1.0935 [0.4225-1.7644]	451	0.875
**MCP-1 (pg/ml)**	48.9825 [42.8158-55.1491]	47.6130 [29.6643-65.5617]	453.50	0.919
**Fractalkine (pg/ml)**	1.8123 [0.7074-2.9173]	0.4117 [0.2669-0.5565]	386	0.256
**VEGFA (pg/ml)**	31.1324 [24.5086-37.7563]	30.8017 [16.9223-44.6811]	442	0.810
